# Osteobiography of a seventh-century potter at the Oupan kiln, China by osteological and multi-isotope approach

**DOI:** 10.1038/s41598-019-48936-1

**Published:** 2019-08-28

**Authors:** Bing Yi, Jinglei Zhang, Botao Cai, Zhongyun Zhang, Yaowu Hu

**Affiliations:** 1 Key Laboratory of Vertebrate Evolution and Human Origins of Chinese Academy of Sciences, Institute of Vertebrate Paleontology and Paleoanthropology, Chinese Academy of Sciences, Beijing, 100044 China; 2 Department of Archaeology and Anthropology, University of Chinese Academy of Sciences, Beijing, 100049 China; 3 School of History, Nanjing University, Nanjing, 210023 China; 4 Institute of Cultural Relics and Archaeology of Anhui Province, Hefei, 230061 China; 5 Anhui Museum, Hefei, 230000 China; 6 Institute of Archaeological Science, Fudan University, Shanghai, 200433 China

**Keywords:** Bone, Stable isotope analysis

## Abstract

In recent years, the reconstruction of individual life history by the multi-isotope analysis of different skeletal elements has become an active topic in bioarchaeological field. However, most studies focus on the persons with high social status and none cares for craftsmen with low social status. In this study, we undertook a comprehensive analysis on a human skeleton buried in the Oupan kiln, Anhui, China to recover his osteobiography. The archaeological context and dating result (534–644 cal. AD) indicate that he might be a potter at the kiln during the Sui and early Tang Dynasty, characteristic of low social hierarchy. The osteological investigation suggests that he had abnormal vertebrae related to long-term physical labor. In general, the isotopic data demonstrate that he mainly consumed C_3_(wheat, beans)/C_4_(millets)-based terrestrial foods. The isotopic (C, N) profiles of dentin sections and isotopic data (C, O) of bone apatite and teeth enamel indicate that he had experienced dramatic dietary changes and/or several migrations throughout the childhood and adulthood. His turbulent life trajectory was highly relevant to his identity and low social status. Our study provides a pilot insight into the life history of craftsmen who was generally overlooked in archaeological, historic and anthropological research.

## Introduction

Since the pioneering research in the late 1970s, stable isotope (C, N) analysis of human skeletons has been widely applied as a routine method for discussing important archaeological issues related to paleodietary reconstruction in bioarchaeological field^1^. In general, the foods revealed by stable isotope analysis of bulk collagen or apatite from humans (animals) bones or teeth only reflect the information on the average diets before their death lasting for at least 5 (ribs) or 10 (femurs) years or during the childhoods (teeth), depending on what skeletal elements are used for analysis. In recent years, the research interests have moved on from the scale of human populations to that of the individuals, trying to reconstruct individual life history or read the individual osteobiography by multi-stable isotope analyses of various skeletal elements. For example, the direct comparison on the isotopic data of human limbs and ribs can get the information on the dietary shift and human mobility due to the fact that the limb and rib have different turnover rates^2–5^. Especially, the method of sequential sampling of tooth dentin recently developed provides valuable information on the high-resolution dietary variations during the childhoods, including the breastfeeding and weaning practices, for the teeth are formed when the individual is young and never remodel since full formation^6–11^. However, compared to those concentrating on the king or nobles or the person with high hierarchy^10,12–16^, studies focusing on the commonality is quite scarce^9,17^.

China, the name of the country, is also the name card of Chinese porcelain. Various and brilliant ancient ceramics have been found extensively in Chinese mainland and are always regarded as one of the best representatives of Chinese civilization^18,19^. However, the life, health and stress of the people producing those ceramics during the prehistoric and historic era have been extremely overlooked for long time in archaeological and historic records, given the fact that the craftsmen in ancient China society are in general related to low social status^20^.

**Figure 1:**
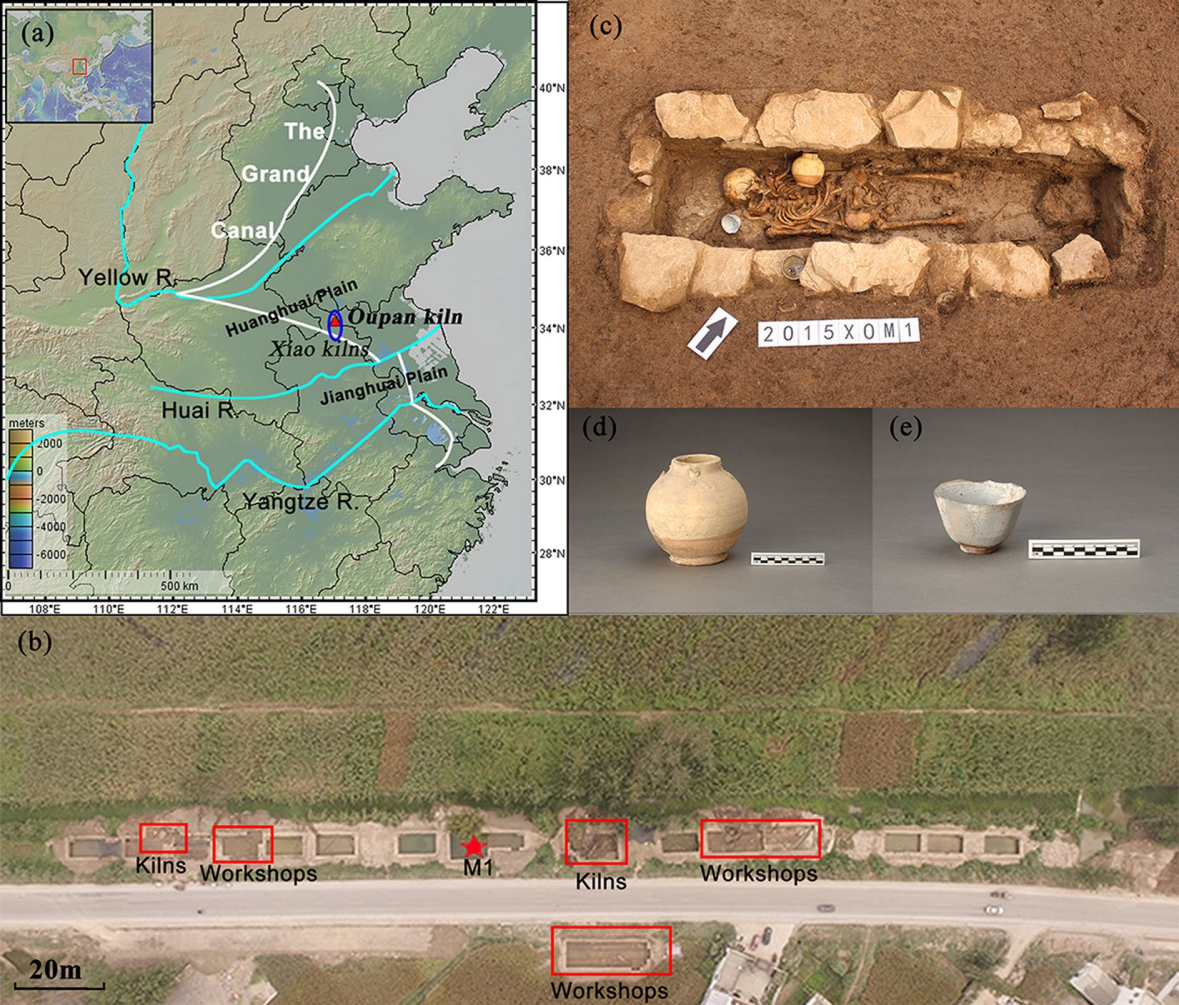
(**a**) Location of main sites mentioned in the text: Oupan kiln (▴), distribution of Xiao kilns (○); (**b**) Location of the tomb (★) and the kilns and workshops (□); (**c**) The human skeleton buried in an extended supine position; (**d**) A yellow-glazed four loop-lugs from the tomb; (**e**) A green-glazed cup from the tomb. Maps were created with software Geo Map v 3.6.10 (http://www.geomapapp.org)^86^. Photograph reproduced with the permission of excavators (the third and fourth authors) of Oupan kiln.

In the Sui and Tang Dynasty, the imperial kiln system had not been formally formed^21^ and the porcelain was mainly produced by the private commercial workshops^22^. The white-glazed porcelain was mainly produced in the north and green-glazed porcelain in the south^19,23^. The procedure of making porcelain is complicated, including selecting raw materials, molding, firing, and glazing, which requires a number of skilled potters^20,24^. Briefly, the potters had a relatively low status^20,25^ and few were mentioned in the historical documents. Here, we made a pilot study on the life history reconstruction of an individual recovered at the Oupan kiln, Xiao County, Anhui Province, China (Fig. 1), during the Sui and Tang Dynasty (581–907 AD), through multi-stable isotope (C, N, O) analysis of diverse skeletal elements including bones and teeth. AMS-^14^C dating of human bones was undertaken to test the possibility of the date coincidence of the individual and the kiln. In addition, osteological observations were also made, trying to identify his pathological conditions which might have been related to his occupation. We believe that our research would shed light on unraveling the osteobiographical secrets hidden in human skeletons on his identity, weaning practice, mobility, health and stresses in his life.

## Methodology of Stable Isotope Analysis

The stable isotope ratios from human skeletal tissues have been widely applied to reconstruct human diets and movements according to principle “You Are What You Eat”^26^. Plants using different photosynthetic pathways (such as C_3_ and C_4_ plants) and in marine and terrestrial ecosystems have different δ^13^C values, and these isotopic variations can also be reflected in the tissues of their consumers including herbivores and carnivores preying on the above herbivores^27–29^. It is generally believed that the carbon in apatite come from the whole diet (carbohydrate, lipids and protein) and that in collagen is mainly derived from the protein in the diet^30^. The carbon isotopic enrichment from the diets to collagen and apatite is about 5‰ and 12‰ for humans respectively^27,29,31^. Recently, the isotopic spacing between collagen and apatite (Δ^13^C_ap–coll_) have been suggested for determining the contribution of C_3_ or C_4_ energy sources to the diets more precisely^32^. The δ^15^N values between the consumer’s tissue and diet are gradually enriched along the food chain by about 3–5‰, given the trophic effect^33,34^. δ^15^N values in the organism are also distinguishable among the terrestrial, freshwater and marine ecosystems due to their various food chains^35^. Thus, the δ^15^N values can reflect the protein sources in the diets and are used for estimating the trophic level^36^. However, it should be pointed out that they are also influenced by many factors such as aridity, salinity, nutritional or physiological stresses^37–40^. In addition, the δ^18^O values in apatite trace back the δ^18^O values of the body water sources, including drinking water, inhaled vapor water and food sources^41^, which is directly relevant to local precipitation and temperature. Therefore, oxygen stable isotope ratios of apatite can provide valuable information on human mobility^41^.

Given the fact that the skeletal elements have different turnover rates, the stable isotope values can stand for the diets and residence at various stages throughout human life. Since bones constantly remodel through lifetime, stable isotope values in bone collagen only mirror the averaged diets before the individual’s death, such as the femurs and ribs, reflecting the diets about 10 years and 2–5 years before death respectively^3,4,42^. As teeth don’t remodel since its formation, the isotopic values in enamel apatite and dentin collagen will record the diets and residence during the periods of tooth development. Therefore, diachronic changes of isotopic values during childhood and adolescence can be revealed by sequential sampling of dentin collagen, considering the incremental growth of teeth from birth to about 20 years of age among the different types of teeth^43,44^. At last, by combining multi-tissue and multi-isotope analyses, dietary and mobile information throughout the lives on individual scale can be reconstructed^12,16,45,46^.

## Archaeological Context

The Xiao kilns named by the Xiao County, Anhui, China (Fig. 1), famous private commercial kiln assemblages in Huai River area, are initially founded in the Sui Dynasty (581-618AD) and ended in the Yuan Dynasty (1271-1368AD)^47,48^. They belong to the gathering place for porcelain exchange between the north and south and deeply affected by both southern and northern porcelain patterns, owing to its unique geographic location close to the Grand Canal (Fig. 1) during the Sui-Tang Dynasties (581-907AD)^48,49^. The Oupan kiln (歐盤窯) (N 34°08′30.64″, E117°02′56.46″), one branch to the Xiao kilns, is located in Baitu Town, Xiao County, Suzhou City, Anhui Province, China and in the Huanghuai Plain between the Yellow River and Huai River valleys geographically (Fig. 1).

From May to October, 2015, a rescue archaeological excavation was jointly conducted by the institute of Cultural Relics and Archaeology of Anhui Province and the Xiaoxian Museum at the area of the Oupan kiln^48^. So far, the area of 1,100 m^2^ has been fully disclosed and nearly 90 cultural remains have been discovered, including 6 kilns, 4 pools, 15 workshops, 1 stove, 50 pits, 7 ditches, 2 pillar holes, and 1 tomb. In about 10,000 artifacts, part of remains such as columns, rods, supports, pads, branch nails, washers, saggers, and so on, were highly relevant to firing the porcelain. The products inside the kiln were mainly composed of green-glazed porcelain as well as a small amount of white-glazed porcelain. The types were mostly bowls and high-footed plates^48^. It was mainly used from the Sui Dynasty (581-618AD) to the glorious age of Tang Dynasty (618–755 AD) according to the preliminary topological analyses of the porcelain^48^. Recent botanical analysis through plant flotation at the Oupan kiln found that the main crops were composed of millets (*Setaria italic* and *Panicum miliaceum*), wheat (*Triticum aestivum*), barley (*Hordeum vulgare*), sorghum (*Sorghum bicolor*), rice (*Oryza sativa*) and beans (*Glycine max*), among which the foxtail millet and wheat were dominant with the beans being second^50^. Unfortunately, no animal remains were found.

The only tomb (M1) was found within the center of those kilns (Fig. 1). It was a rectangular stone chamber tomb, containing a human skeleton buried with an extended supine position. Some slabs around the tomb were broken and the fibula and tibia of the individual were lost (Fig. 1), probably caused by the artificial damage during later periods. Two pieces of funerary porcelain were found, which were illustrated in Fig. 1. According to the geological layer of the tomb and the typological analysis of funerary porcelain, this tomb are likely dated to Sui and the early Tang Dynasty^48^.

## Results

### Radiocarbon dating

According to Table 1, the calibrated date of the human individual is 534–644 cal. AD (2σ). Therefore, in combination with the archaeological context, the human individual must have lived during the Sui and early Tang Dynasty.

**Table 1:** Radiocarbon age for the skeleton in this study.

Lab Code	Sample type	^14^C date (BP)	Calibrated Age (cal AD)	
			1σ (68.2%)	2σ (95.4%)
Beta-497624	Bone	1490 ± 30	549–605 (68.2%)	534–644 (92.2%)
				472–486 (2%)
				436–446 (1.2%)

### Osteological results

#### Sex and age determination

**Figure 2:**
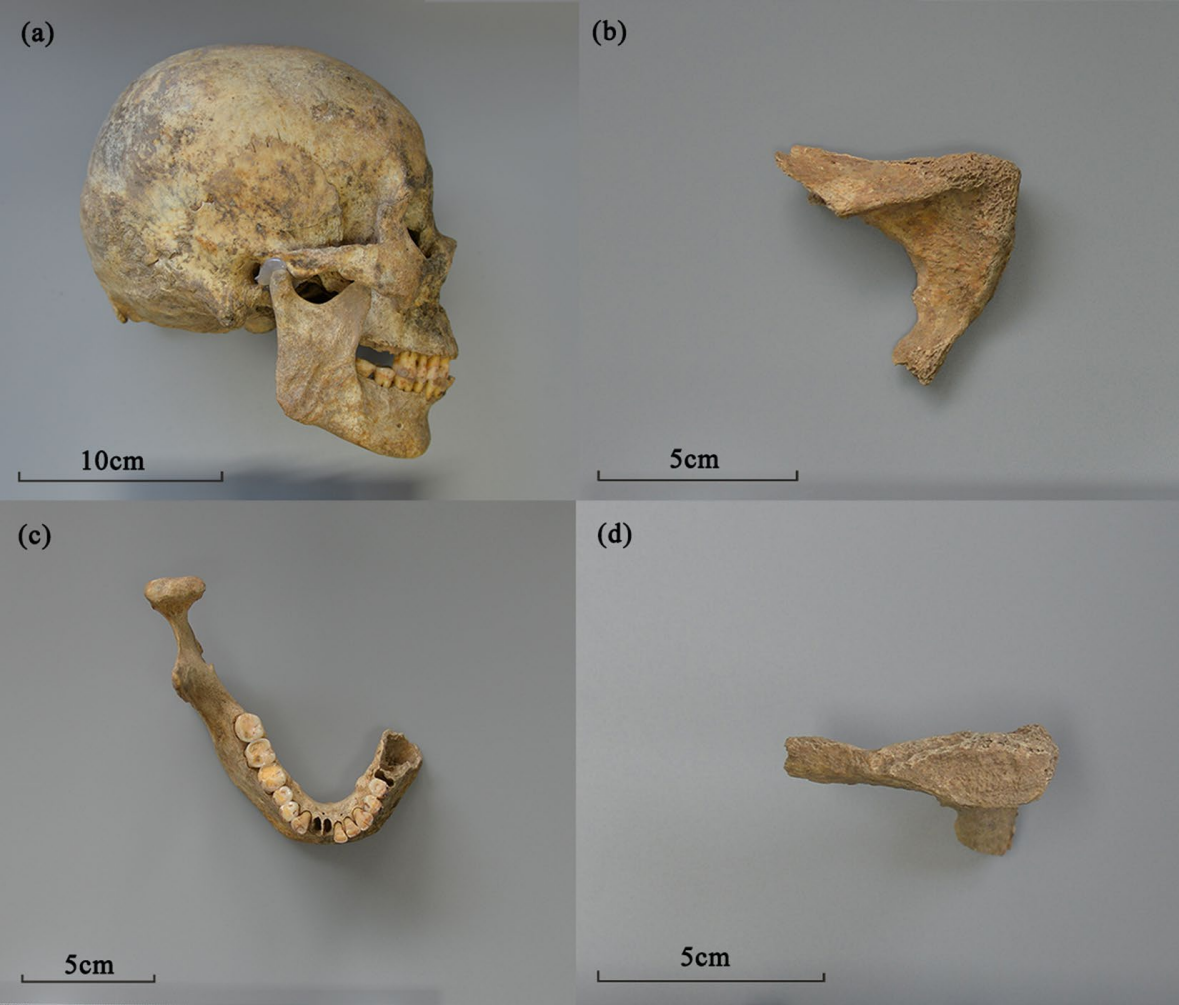
Determination of age and sex (**a**) Skull characteristics; (**b**) Subpubic angle; (**c**) Teeth attrition stage H; (**d**) Pubic symphysis surface phase 5.

This individual was characterized by robust and thick crania, heavier temporal line, more inclined frontal bone, prominent superciliary arch, eversion of the angle of mandible, narrow greater sciatic notch, small subpubic angle, strong limbs and *etc*., suggesting a male (Fig. 2). The age was estimated to be about 45 years old, evidenced by the teeth attrition stage H (40–45 yrs) and pubic symphysis surface phase 5 (~45 yrs) (Fig. 2).

#### Pathological conditions

**Figure 3:**
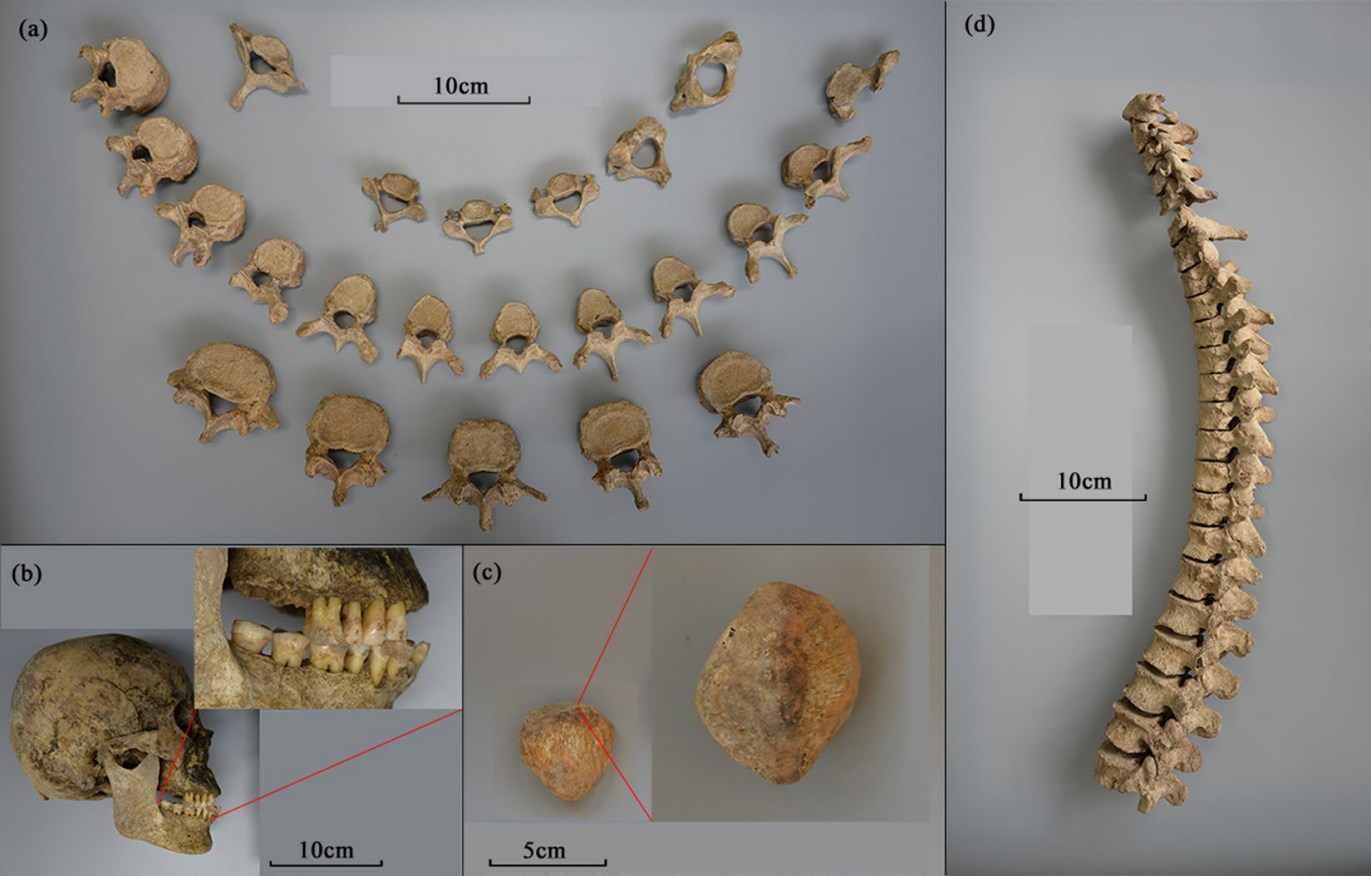
The pathological conditions of this individual: (**a**) Marginal osteophytes on vertebral columns; (**b**) Roots exposure and alveolar resorption indicative of periodontal disease; (**c**) Marginal osteophytes and bone spurs on the right patella; (**d**) Abnormal vertebral curvature. Note: C6 was lost.

Marginal osteophytes on vertebrae were diagnosed (Fig. 3). Osteophytes are manifestations of degenerative joint disease (DJD), or osteoarthritis (OA)^51^. An important cause of this disease is aging. However, behavioral factors, especially mechanical load, can accelerate such degenerative lesions^52^. According to the stages of the severity of vertebral osteophytosis recorded in Rojas‐Sepúlveda *et al*.^53^ and Hou *et al*.^54^, the grades of marginal osteophytes on these vertebrae were listed in Supplementary Table S1. The osteophytes were generally moderate and no ankylosing spondylitis was observed. Besides, vertebrae of T12 and L2-L4 showed a wedge change, and the curvature of the thoracic and the lumbar vertebrae displayed abnormality, indicating the humpback of this individual (Fig. 3).

Knee osteoarthritis, related to aging, occupational knee bending and physical labor and *etc*.^55^, was also confirmed by marginal osteophytes and bone spurs on the right patella (Fig. 3). However, due to burial damages, the actual length of the bone spurs cannot be measured.

In addition, periodontitis was identified by marked exposure of the roots (3.4~7.8 mm) and alveolar bone resorption (1/3 ~1/2 of the root) (Fig. 3)^56,57^. This disease is caused by pathogenic bacteria in the dental plaque^51^, and has a high prevalence among people who are predominantly on a plant diet with large quantity of carbohydrate^58^.

### Isotopic data from bone and dentin collagen and enamel

**Figure 4:**
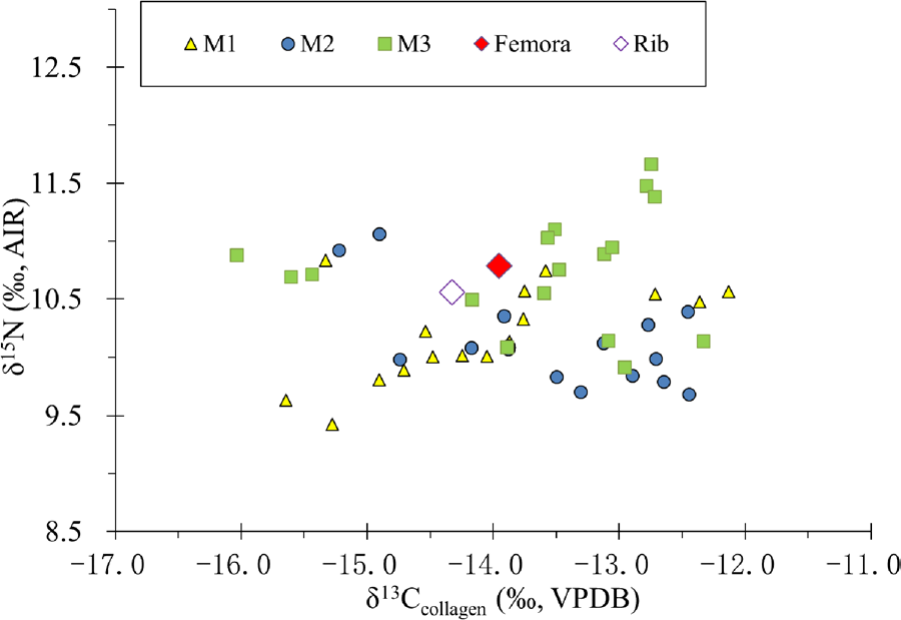
Scatter plot of δ^13^C and δ^15^N values of collagen for femur, rib and dentin serials.

As seen in Fig. 4 and Supplementary Table S2, femur (δ^13^C_femur_: −14.0‰; δ^15^N_femur_: 10.8‰) and rib (δ^13^C_rib_: −14.3‰; δ^15^N_rib_: 10.6‰) have nearly identical isotopic values, indicating a stable diet for the last 5 or 10 years before death. In addition, the isotopic results suggest the diet was mixed with C_3_/C_4_-based terrestrial protein. Compared to those from the rib and femur, the isotopic results from serial dentin collagen display substantial variation. The δ^13^C_dent_ values range from −16.0‰ to −12.1‰ and δ^15^N_dent_ values from 9.4‰ to 11.7‰ respectively. This demonstrates quite large dietary shifts during the childhood and adulthood of this individual.

**Figure 5:**
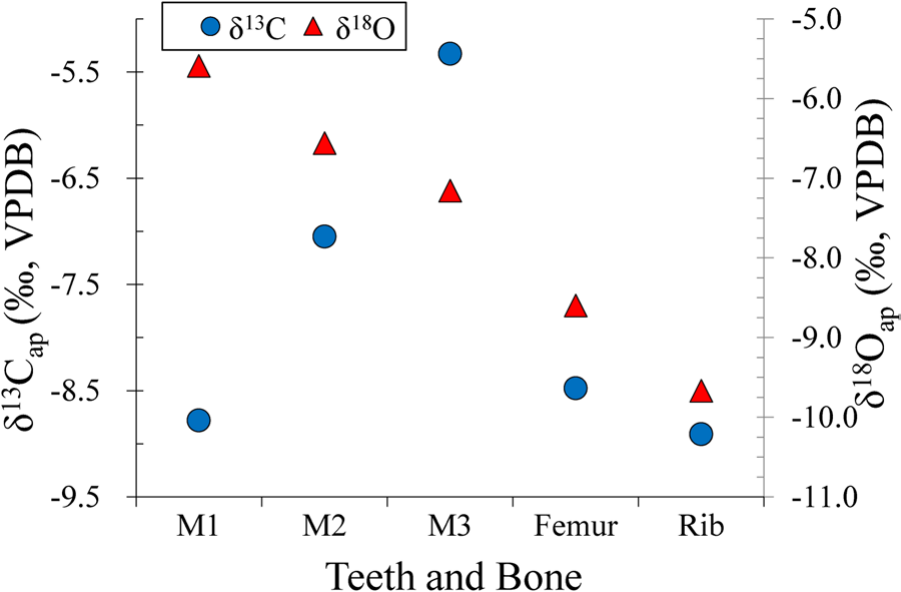
Scatter plot of δ^13^C and δ^18^O values from teeth enamel and bone apatite.

Isotope (C, O) results of enamel and bones are plotted in Fig. 5 and listed in Supplementary Table S3. The δ^13^C values also indicate that the individual consumed C_3_/C_4_ mixed foods if the isotopic enrichment from the diet to apatite (12‰)^31^ is used. It is easily seen that there is an increase of δ^13^C_enamel_ values from −8.8‰ to −5.3‰ and a decrease of δ^18^O_enamel_ values from −5.6‰ to −7.2‰ throughout the individual early life. This pattern possibly suggests the frequent shifts of residence and diets during the early stage of individual’ life history, confirming the results based on the isotopic results from dentin collagen. Similarity of isotopic data from femur and rib can be seen in Fig. 5, reconfirming the above inference from the isotopic (C, N) data. Nevertheless, it should be noted that there is a large isotopic difference between LM_3_ and bones (rib and femur) in Fig. 5, implying that he might have migrated elsewhere. This will be discussed in more detail in the following sections.

## Discussions

It is necessary to discern the human identity before reconstructing the life history of the individual, as the life history might have been deeply influenced by his occupation and social status. Several criteria were put forward as follows, trying to identify the human status.

This tomb is the only one located within the kiln and near the workshop area (Fig. 1). Besides, the typological analysis of the two funerary porcelain in the tomb showed that they were similar to those found in the kiln^48^. Most importantly, the date of the individual on the basis of radiocarbon dating in this study is well located within the chronological range when the kiln was widely used. Therefore, we can infer that there was a close connection between the individual and the kiln.

His poor social status is evidenced by the existence of the two porcelain objects in the tomb and simple and small tomb layout. Additionally, degenerative joint disease (DJD) or osteoarthritis (OA) was observed in the individual’s skeleton (Fig. 3). Generally, DJD is considered to be caused by long-term repeated activity besides aging factor^59–62^, which is highly related to the human occupation. If the person is engaged in long-term bending and weight-bearing activity, resulting in a constant pressure on the joints, some degenerative joint diseases such as vertebral osteophytes and knee osteoarthritis may occur^58^, which were also present here (Fig. 3). Long-term physical labor may have even led to his abnormal vertebral curvature (Fig. 3).

In summary, we conclude temporarily that the individual probably worked at the kiln for a long time as a potter and was simply buried here after he died, based on archaeological, anthropological and dating evidence.

Since the Neolithic period (~8000–4000BP), the millet and rice mixed agriculture had formed in the Huanghuai Plain^63,64^. According to ancient Chinese historical documents, such as *Sishizuanyao* (四時纂要)^65^ and *Xintangshu* (新唐書·食貨志)^66^, the agriculture in Huanghuai Plain during early Tang Dynasty included cultivation of millets (a C_4_ crop), wheats (a C_3_ crop), and rice (a C_3_ crop), but varied to some extents in the north and in the south^67–70^. On the other hand, the Grand Canal connecting the Yellow River, the Huai River, the Yangtze River and the Qiantang River, resulted in fast and smooth exchange of goods between the north and the south^22^. Thus, rice planted widely in the south was transferred to the north where was the economic and political center during the Sui and Tang Dynasties^71^. Millets were still the staple food for residents in the north of the Huai River while rice was mainly provided for the upper classes in the north according to the historical records such as *Sishizuanyao* (四時纂要)^65,71^.

On the basis of the historic documents and crop remains at the kiln^50^, we deduce that the carbon isotopic signals from the human collagen and enamel apatite could both reflect the consumption of millets (C_4_) and wheat/beans (C_3_)-based foods. Furthermore, the carbon isotopic spacing (Δ^13^C_ap–coll_) between the collagen and apatite offers an effective way to reveal the energy sources (carbohydrate) more precisely^72^.

The mean δ^15^N_dent_ values from three teeth dentin and the δ^15^N values from bone collagen in Table 2 are similar (~10‰), but the δ^13^C_dent_, δ^13^C_ap_, and Δ^13^C_ap–coll_ values are different. The linear increase of δ^13^C_dent_, δ^13^C_ap_ and Δ^13^C_ap–coll_ values from LM_1_ to LM_3_ (Table 2) strongly indicates that the consumption of millets were enhanced throughout the early stage of his life. On the other hand, lower δ^13^C_dent_ and δ^13^C_ap_ values and smaller Δ^13^C_ap–coll_ values from the rib and femur than those from LM_3_ teeth indicate that the human individual consumed more C_3_ foods (wheats/beans) before his death.

**Table 2:** Carbon isotope values from the enamel apatite and dentin collagen, and mean nitrogen isotope values of dentin collagen.

Type	δ^13^C_ap_ (‰)	Approximate age of crown/bone development (in years)	Mean δ^13^C_den_ (‰) with the ages corresponding to crown development	Δ^13^C_ap-coll_	Mean δ^15^N_den_ (‰) and δ^15^N_bone_ (‰)
LM_1_	−8.8	0.3–3.5	−15.3	6.5	10.2
LM_2_	−7.0	2.5–8.5	−14.0	7.0	10.1
LM_3_	−5.3	8.5–14.5	−13.1	7.8	10.8
Femur	−8.5	~10 years before death	−14.0	5.5	10.8
Rib	−8.9	~2–5 years before death	−14.3	5.4	10.6

Note:

1. The mean δ^13^C_den_ values refer to the isotopic average of dentin sections with the age corresponding to the crown of each tooth.

2. The mean δ^15^N_den_ values refer to the isotopic average of whole dentin sections of each tooth.

**Figure 6:**
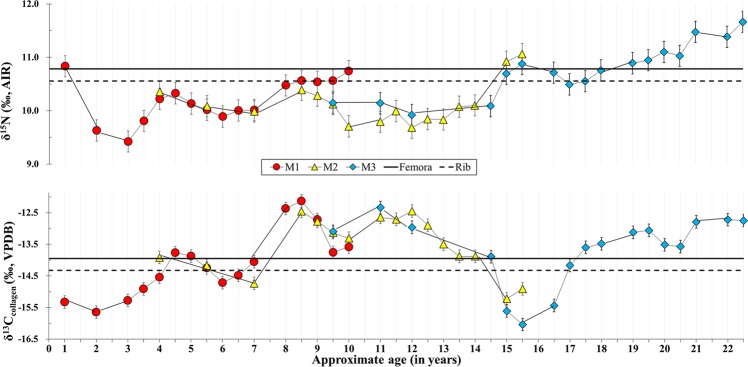
Nitrogen and carbon isotopic profiles of teeth serial sections.

Moreover, the isotopic profiles of teeth sections as well as femur and rib in Fig. 6 can reconstruct most of his life history before the individual’s death. The similarity of isotopic data between femur and rib in Fig. 6 indicates that this male individual had a relatively stable life before his death. However, large isotopic variation in his teeth show that his diet varied greatly in his childhood and early adulthood.

The weaning of this individual might occur around 2 years old, which is evidenced by sharp decrease of δ^15^N_dent_ values from the age of 1 to 2–3 and δ^13^C_dent_ values at the age of 2. It is noted that the millets-based foods, including millets and/or millets byproducts-consuming animals, contributed substantially to the infant’s diets. During the age of 3–9, he had more accesses to millets-based foods, resulting in the gradual increase of δ^13^C_dent_ values in Fig. 6. Afterwards, he had a steady life during the age of 9–14, which is evidenced by the minor shift of isotopic data. It is noteworthy that during the age of 14–16, an opposite profile of δ^13^C_dent_ and δ^15^N_dent_ value variations occurs in Fig. 6. This isotopic pattern is much likely caused by the nutritional or physiological stresses^6,38^ during his adolescence. Since the age of 16, the increase of δ^13^C_dent_ and δ^15^N_dent_ values has been observed in the profile of the third molar, indicating that the individual consumed more millets-based protein again.

Why did the individual’s diet shift all the time during his early life? It could be likely due to the dietary preferences in different ages, but also likely caused by human mobility. Here, we analyze the oxygen stable isotope values in teeth and bones to test the above hypothesis. The δ^18^O values of meteoric water generally decrease with the rise of latitude, *i*.*e*. latitude effect^41^. As a consequence, higher δ^18^O values in human bone (teeth) apatite are usually observed in lower latitude than in higher latitude, even though there are annual and seasonal variations of δ^18^O values to possibly influence their spatial distribution^73^. The continuous decrease of δ^18^O values from LM_1_ to femur and rib is seen in Fig. 5, implying that the individual migrated all his life. Given the facts of widely cultivated millets in the North China and the negative correlation of *δ*^13^C and δ^18^O values in the three molars, it seems that this individual could have moved northwards and had more accesses to millet-based foods during the early stage of his life. This is also confirmed by the gradual increase of mean *δ*^13^C values from the dentin collagen in Table 2. He only settled down and lived a relatively stable life 10 years ago before his death, which is evidenced by the similarity of isotopic data (C, N, O) between the rib and femur (Fig. 5 and Fig. 6).

The Oupan kiln is located in a transitional zone of the north and the south producing the white-glazed porcelain and green-glazed porcelain respectively, where most of green-glazed porcelain and small amount of white porcelain was manufactured^49^. Making large quantity of green-glazed porcelain should require skillful potters who originate from the south and are familiar with the procedure. Thus, it is plausible that the potter studied here was recruited from the south and worked at the kiln for long time.

However, considering the difficulty to determine the “local” isotopic range by δ^18^O values alone^41^, we can definitely tell that the variations of δ^18^O values here are merely derived from human movement. Sr isotope analysis of human teeth and combination with oxygen isotope values will help to clarify it more in the future.

In brief, the isotopic analysis of the bones and teeth from this individual truly recorded the unsteady life and nutritional or physiological stresses of the potter working at the Oupan kiln during the Sui and Early Tang Dynasty. These isotopic signals and the pathological conditions of his skeleton are consistent with his low social status and demonstrate a real life history of a common person that is always neglected before.

The Chinese poem written by Fu Du, *the wine and meat was stink in the rich family while the froze bones were present by the road* (朱門酒肉臭,路有凍死骨), described vividly the dramatic difference of the lifestyles between the rich and the poor during the Tang Dynasty. Despite abundant literature relevant to Sui-Tang histories, there are little records on craftsmen, the creators of social material civilization. Here, our study provides a good case to reveal the life trajectory of a potter and verify some records in historic literature.

The Chinese medical literature, such as Qianjinfang (千金方) by Sun in the Tang Dynasty^74^, Youyouxinshu (幼幼新書) by Liu in the Song Dynasty^75^, Yixuerumen (醫學入門) by Li in the Ming Dynasty^76^, mention the breastfeeding and weaning practices of infants in the past. They recommend that breastfeeding should not be too long, i.e., not more than 2–3 years old, and that it is suitable for feeding infants porridge after half a year. Millet porridge was commonly provided for the infants in the Tang Dynasty as special nutrition^71^. In this study, the cessation of weaning of this individual was completed around at the age of 2 and millets contributed substantially to his diet during the breastfeeding and weaning process. Thus, our study is consistent to the records in the historic literature and illustrates directly the importance of feeding millets to infants that can trace back to the Late Neolithic^11^.

The reconstruction of individual life history by stable isotope analysis offers a good means to trace back the life trajectory of the individual that cannot be obtained from the literature. In this study, the stable isotope analyses of the individual’s bones and teeth clearly demonstrate that he lived a volatile life and possibly migrated several times spanning his lifetime. In the meantime, he suffered from physiological or nutritional stresses at the ages of 14–16. His unsteady life and health might be highly related to his low social status and potter identity.

## Conclusion

The human skeleton discovered at the Oupan Kiln, Xiao Country, Anhui Province, China provides an opportunity to investigate the life history of a potter with low social status during the booming period of the porcelain in the Sui-early Tang Dynasty by osteological and multi-stable isotope analyses. The osteological investigation showed that this male suffered from a heavy stress on the vertebrae and knee. The isotopic data of bones (femur and rib), enamel and dentin serials were indicative of substantial dietary fluctuation and possible frequent migration throughout his life. This is consistent with his identity as a potter. Therefore, our study here illustrates a pilot study to reveal the osteobigraphy of craftsmen in the prehistoric or historic era which has been overlooked before. We hope that similar studies focusing on the life history reconstruction of craftsmen as the founders of human civilization and creators of exquisite artifacts should be undertaken more in the future.

## Materials and Methods

### AMS-^14^C dating of human bones

One piece of the individual femur was cut and sent for AMS-^14^C dating in Beta Analytic. The pretreatment and measurement protocols can be found in the website: https://www.radiocarbon.com/pretreatment-carbon-dating.htm#Acid. and https://www.radiocarbon.com/beta-radiocarbon-lab.htm. respectively. The result was shown in Table 1.

### Osteological observations

Osteological observations on the individual, including the determination of age and sex and identification of pathology, were undertaken in the physical anthropological lab by Dr. Jinglei Zhang from School of History, Nanjing University by standard anthropological^77,78^ and pathological^51,57^ methods. Some of pathological bones were shown in Fig. 3.

### Multi-stable isotope analysis of the human skeleton

Here, various skeletal elements of the individual, listed in Supplementary Table S4, were selected on purpose for stable isotope analysis, as they had different turnover rates and can represent the different stage in human life. Age estimation of development for each tissue was based on Cox & Sealy^3^, Parfitt^4^, Hedges *et al*.^42^, and Beaumont and Montgomery^79^.

#### Sequential sampling of teeth

Incremental sampling of the teeth listed as above was prepared at the Key Laboratory of Vertebrate Evolution and Human Origins of the Chinese Academy of Sciences, Institute of Vertebrate Palaeontology and Palaeoanthropology, Chinese Academy of Sciences, using the method 2 proposed by Beaumont *et al*.^80^. Dentine collagen was prepared using the modified protocols proposed by Richards and Hedges^81^. Samples were cleaned first by mechanically removing surface debris and rinsing in an ultrasonic bath. Then, following manual removal of the enamel, teeth were demineralized in a 0.5 M hydrochloric acid (HCl) solution at 4 °C for ~2 weeks, with the acid refreshed every 24–48 h, and then were rinsed at least three times in deionized water, soaked in a 0.125 M sodium hydroxide (NaOH) solution at 4 °C for ~20 h and rinsed to neutrality again. Serial samples of demineralized dentin were cut at 1 mm interval transversely. Subsequent to demineralization and sectioning, dentin serial samples were gelatinized at 70 °C in pH = 3 hydrochloric acid (HCl) solution for 24–48 hours. Finally, they were filtered and freeze-dried. Age estimation of each section was based on the method of Beaumont and Montgomery^79^.

#### Bone Collagen preparation

Bone collagen preparation was also followed the modified protocols proposed by Richards and Hedges^81^. The processing of bones was the same as that of teeth, except for the sequential sampling.

#### Bone and Tooth apatite preparation

Bone and dental enamel powders were prepared according to modified method of Lee-Thorp *et al*.^72^. Powder samples were drilled by a diamond-tipped dental burr. After collecting about 10 mg powder, the samples were soaked in a 50% sodium hypochlorite (NaOCI) solution at 4 °C for about 48 h, rinsed thoroughly with deionized water, leached at 4 °C for 24 h in 1 M acetic acid solution, rinsed to neutrality again, and then freeze-dried.

Teeth are thought to be the most resistant to diagenetic alternation in the vertebrate body and widely used as best candidate for stable isotope analysis^82^. However, bone apatite is less resistant than tooth enamel and whether it is suitable for isotopic analysis is still in debate^83^. Here, the apatite preservation of human femur and rib were further analyzed by the methods of X-ray Diffraction (XRD) and Fourier Transform Infrared Spectroscopy (FTIR) to assess the integrity of apatite crystallinity as seen in Supplementary Fig. S1 and Supplementary Fig. S2. Based on the bone apatite crystallinity indexes, PCI and BPI (Supplementary Table S5), and bone collagen quality (Supplementary Table S2), we believe that they retain the *in vivo* isotopic signature of apatite carbonate and good for isotopic analysis (see the detailed discussion in Supplementary Methods).

#### *Measurements of elemental (C*, *N) contents and stable isotope values (C*, *N) of collagen and stable isotope (C*, *O) values of apatite*

Collagen samples were packed into tin boats (~0.5–1 mg) for carbon and nitrogen isotope analyses. Samples were measured at the Archaeological Stable Isotope Laboratory (ASIL), the Department of Archaeology and Anthropology at the University of the Chinese Academy of Sciences, using the Isotope Ratio Mass Spectrometer (IsoPrime 100) coupled with the elemental analyzer (Elementar Vario Pyrobe). The isotopic results were expressed as δ in parts per mil (‰), normalized to the internationally defined standards for carbon (VPDB) and nitrogen (AIR) respectively. The international standards were Acetanilide, IAEA-600, IAEA-N-2, IAEA-CH-6, USGS 40 and USGS 41, and a collagen lab standard (δ^13^C value of −14.7 ± 0.2‰ and δ^15^N value of 7.0 ± 0.2‰) for isotopic calibration. Analytical uncertainty is <±0.2‰ for both δ^13^C and δ^15^N values.

Prepared enamel powder samples were packed in sealed glass tubes, placed in a hot block at 80 °C and flushed with high-purity helium for 120 seconds for each sample, then were added into ~0.6 ml of 100% orthophosphate acid (H_3_PO_4_), reacted at 80 °C for 1 h. Carbon and oxygen isotope analyses were measured by an Isoprime 100 IRMS coupled with a multi-flow system at the Archaeological Stable Isotope Laboratory (ASIL), Department of Archaeology and Anthropology, University of Chinese Academy of Sciences, reported as δ in parts per mil (‰) relative to the international standards VPDB using IAEA CO-8 and NBS 19 for isotopic calibration and NBS 18 for monitoring the instrument stability. Analytical uncertainty is better than ± 0.2‰ for both δ^13^C and δ^18^O values.

It is seen in Supplementary Table S2 that both of the bone samples including rib and femur, and 49 dentin serial sections from first molar, second molar and third molar of this individual, yielded good quality collagen for stable isotope analysis with atomic C/N ratios ranging from 2.9 to 3.6^84^, nitrogen concentrations (%N) and carbon concentration (%C) above 4.8% and 13% respectively^85^.

## Supplementary information

Supplementary Information
