# Supplementary material for: Osteobiography of a seventh-century potter at the Oupan kiln, China by osteological and multi-isotope approach
Source: Sci Rep. 2019 Aug 28;9:12475. doi: 10.1038/s41598-019-48936-1 (PMC6713732; doi:10.1038/s41598-019-48936-1)
Supplement: Supplementary file 1 — Supplementary Information [file 41598_2019_48936_MOESM1_ESM.doc]

**Osteobiography of a seventh-century potter at the Oupan kiln, China by osteological and multi-isotope approach**

Bing Yi^1,2^, Jinglei Zhang^3*^, Botao Cai^4^, Zhongyun Zhang^5^, Yaowu Hu^6,1,2*^

*^1^Key Laboratory of Vertebrate Evolution and Human Origins of Chinese Academy of Sciences, Institute of Vertebrate Paleontology and Paleoanthropology, Chinese Academy of Sciences, Beijing 100044, China*

*^2^Department of Archaeology and Anthropology,* *University of Chinese Academy of Sciences, Beijing 100049, China*

*^3^School of History, Nanjing University, Nanjing 210023, China*

*^4^Institute of Cultural Relics and Archaeology of Anhui Province, Hefei 230061, China*

*^5^Anhui Museum, Hefei 230000, China*

*^6^Institute of Archaeological Science, Fudan University, Shanghai 200433, China*

* = Address for correspondence:

J. Zhang e-mail: zhangjinglei@nju.edu.cn

Y. Hu e-mail: [ywhu@ucas.ac.cn](mailto:ywhu@ucas.ac.cn)

**Supplementary Information**

Supplementary Methods

Supplementary Table S1. Stages recorded for the manifestations of vertebral degenerative conditions.

Supplementary Table S2. Isotopic results and sample information for all human dentine serials and bones analyzed from the Oupan kiln in Anhui Province, China.

Supplementary Table S3. Isotopic results for enamel and bones apatite analyzed from the Oupan kiln in Anhui Province, China.

Supplementary Table S4. Sample information including skeletal elements, age estimation, and the number of isotopic serial samples analyzed in this study.

Supplementary Table S5. The PCI and BPI of the femur and rib.

Supplementary Fig. S1. X-ray diffraction diagram of femur and rib.

Supplementary Fig. S2. FTIR spectra of femur and rib.

References

**Supplementary Methods**

So far, there have had no well-recognized international criteria for assessing the carbonate preservation in bones apatite. Here we use two methods to test the integrity of bone apatite, which is proven to be effective in diagenetic analysis.

1. The correlation of bone apatite and collagen. In general, bad preservation of collagen can lead to the deterioration of bone apatite as the bone apatite is located within the cavity of the collagen fibers connecting each other [^1^](#_ENREF_1). In our study, the collagen yields, contents of carbon and nitrogen, and the atomic ratios of C/N of collagen extracted from the rib and femur all show that the collagen was well preserved.

2. Crystallinity of bone apatite. Due to the long-term burial and the interaction with the environment, the peaks of apatite from ancient bones in XRD patterns is generally sharper than those from modern bones [^2^](#_ENREF_2), which is observed here too in Supplementary Fig. S1. In addition, the XRD patterns in Supplementary Fig. S1 show that no other minerals than hydroxyapatite were preserved in the rib and femur. On the other hand, the crystallinity indexes, PCI (Phosphate Crystallinity Index) and BPI (Carbonate Phosphate Index) [^3^](#_ENREF_3)observed and calculated in FTIR, are commonly used in the literature to understand the diagenesis of bone apatite. PCI is used to monitor the crystal alternation of phosphate in apatite, which ranges from 2.8 to 3.0 in modern bone apatite, and is acceptable from 3.5 to 4.8 in archaeological bones . And the BPI ranges from 0.29 to 0.82 in modern samples, and from 0.6 to 0.8 in archaeological bones [^6^](#_ENREF_6). It is easily seen that these indexes of the femur and rib in our study are located with the range in Supplementary Table S5.

Summarizing the above, we can say that the bone apatite extracted from the rib and femur suffered from little diagenetic effects.

**Supplementary Table S1.** Stages recorded for the manifestations of vertebral degenerative conditions.

| Stage | Vertebral columns |
| --- | --- |
| 1 | C1- C3; C7 |
| 2 | C4; T1-T6; L5 |
| 3 | C5; T7-T12; L1-L4 |
| 4 | None |

Note: C, T and L are cervical, thoracic and lumbar vertebrae respectively. C6 was lost. Stage 0, no osteophytes; Stage 1, the margin of vertebral body is slightly protruding, but no joint surface contour change or ‘‘lipping’’; Stage 2, lip-shaped osteophytes are formed, but do not exceed 2 mm beyond the margin of the vertebral body; Stage 3, obvious osteophytes are formed, and are 2 mm beyond the margin of the vertebral body; Stage 4, osteophytes on adjacent vertebrae fused, result in ankylosing spondylitis.

**Supplementary Table S2.** Isotopic results and sample information for all human dentine serials and bones analyzed from the Oupan kiln in Anhui Province, China.

|  | | **Sample and section number** | **δ^13^C(‰)** | **δ^15^N(‰)** | **%C** | **%N** | **Atomic C/N** | **Collagen Yield (%)** | **Estimated age in years based on Beaumont & Montgomery (2015)** | | --- | --- | --- | --- | --- | --- | --- | --- | | Femur | -14.0 | 10.8 | 42.3 | 15.6 | 3.2 | 6.5% |  | | Rib | -14.3 | 10.6 | 36.6 | 13.5 | 3.2 | 2.5% |  | | LM_1_-1 | -15.3 | 10.8 | 42.3 | 15.3 | 3.2 |  | 1.1 | | LM_1_-2 | -15.7 | 9.6 | 41.9 | 15.4 | 3.2 |  | 1.9 | | LM_1_-3 | -15.3 | 9.4 | 41.7 | 15.5 | 3.2 |  | 2.7 | | LM_1_-4 | -14.9 | 9.8 | 42.5 | 15.5 | 3.2 |  | 3.5 | | LM_1_-5 | -14.5 | 10.2 | 41.8 | 15.2 | 3.2 |  | 4 | | LM_1_-6 | -13.8 | 10.3 | 41.5 | 15.4 | 3.1 |  | 4.6 | | LM_1_-7 | -13.9 | 10.1 | 42.4 | 15.5 | 3.2 |  | 5.1 | | LM_1_-8 | -14.3 | 10.0 | 41.6 | 15.4 | 3.2 |  | 5.7 | | LM_1_-9 | -14.7 | 9.9 | 41.9 | 15.3 | 3.2 |  | 6.2 | | LM_1_-10 | -14.5 | 10.0 | 42.0 | 15.4 | 3.2 |  | 6.7 | | LM_1_-11 | -14.1 | 10.0 | 42.3 | 15.4 | 3.2 |  | 7.3 | | LM_1_-12 | -12.4 | 10.5 | 41.8 | 15.3 | 3.2 |  | 7.8 | | LM_1_-13 | -12.1 | 10.6 | 42.4 | 15.3 | 3.2 |  | 8.4 | | LM_1_-14 | -12.7 | 10.5 | 41.4 | 15.2 | 3.2 |  | 8.9 | | LM_1_-15 | -13.8 | 10.6 | 42.3 | 15.4 | 3.2 |  | 9.4 | | LM_1_-16 | -13.6 | 10.7 | 41.0 | 14.9 | 3.2 |  | 10 | | LM_2_-1 | -13.9 | 10.4 | 41.9 | 15.4 | 3.2 |  | 4 | | LM_2_-2 | -14.2 | 10.1 | 42.7 | 15.5 | 3.2 |  | 5.5 | | LM_2_-3 | -14.7 | 10.0 | 42.0 | 15.5 | 3.2 |  | 7 | | LM_2_-4 | -12.5 | 10.4 | 42.1 | 15.5 | 3.2 |  | 8.5 | | LM_2_-5 | -12.8 | 10.3 | 41.6 | 15.2 | 3.2 |  | 9.1 | | LM_2_-6 | -13.1 | 10.1 | 43.2 | 15.5 | 3.3 |  | 9.7 | | LM_2_-7 | -13.3 | 9.7 | 42.5 | 15.5 | 3.2 |  | 10.2 | | LM_2_-8 | -12.7 | 9.8 | 41.5 | 15.4 | 3.2 |  | 10.8 | | LM_2_-9 | -12.7 | 10.0 | 43.6 | 15.8 | 3.2 |  | 11.4 | | LM_2_-10 | -12.5 | 9.7 | 42.1 | 15.4 | 3.2 |  | 12 | | LM_2_-11 | -12.9 | 9.8 | 41.6 | 15.4 | 3.2 |  | 12.6 | | LM_2_-12 | -13.5 | 9.8 | 42.3 | 15.5 | 3.2 |  | 13.1 | | LM_2_-13 | -13.9 | 10.1 | 42.5 | 15.3 | 3.2 |  | 13.7 | | LM_2_-14 | -13.9 | 10.1 | 41.8 | 15.3 | 3.2 |  | 14.3 | | LM_2_-15 | -15.2 | 10.9 | 41.9 | 15.3 | 3.2 |  | 14.9 | | LM_2_-16 | -14.9 | 11.1 | 42.5 | 15.4 | 3.2 |  | 15.5 | | LM_3_-1 | -13.1 | 10.2 | 41.8 | 15.5 | 3.2 |  | 9.7 | | LM_3_-2 | -12.3 | 10.1 | 41.8 | 15.5 | 3.2 |  | 10.9 | | LM_3_-3 | -13.0 | 9.9 | 41.7 | 15.5 | 3.1 |  | 12.1 | | LM_3_-4 | -13.9 | 10.1 | 41.7 | 15.4 | 3.2 |  | 14.5 | | LM_3_-5 | -15.6 | 10.7 | 42.0 | 15.4 | 3.2 |  | 15.1 | | LM_3_-6 | -16.0 | 10.9 | 41.6 | 15.3 | 3.2 |  | 15.7 | | LM_3_-7 | -15.4 | 10.7 | 45.9 | 17.0 | 3.2 |  | 16.4 | | LM_3_-8 | -14.2 | 10.5 | 41.6 | 15.4 | 3.2 |  | 17 | | LM_3_-9 | -13.6 | 10.6 | 41.4 | 15.3 | 3.2 |  | 17.6 | | LM_3_-10 | -13.5 | 10.8 | 42.2 | 15.4 | 3.2 |  | 18.2 | | LM_3_-11 | -13.1 | 10.9 | 41.7 | 15.3 | 3.2 |  | 18.8 | | LM_3_-12 | -13.1 | 11.0 | 42.9 | 15.8 | 3.2 |  | 19.5 | | LM_3_-13 | -13.5 | 11.1 | 42.3 | 15.5 | 3.2 |  | 20.1 | | LM_3_-14 | -13.6 | 11.0 | 42.1 | 15.4 | 3.2 |  | 20.7 | | LM_3_-15 | -12.8 | 11.5 | 42.6 | 15.4 | 3.2 |  | 21.3 | | LM_3_-16 | -12.7 | 11.4 | 41.6 | 15.1 | 3.2 |  | 21.9 | | LM_3_-17 | -12.8 | 11.7 | 41.2 | 14.9 | 3.2 |  | 22.6 | |  |  |  |  |  |  |
| --- | --- | --- | --- | --- | --- | --- | --- | --- | --- | --- | --- | --- | --- | --- | --- | --- | --- | --- | --- | --- | --- | --- | --- | --- | --- | --- | --- | --- | --- | --- | --- | --- | --- | --- | --- | --- | --- | --- | --- | --- | --- | --- | --- | --- | --- | --- | --- | --- | --- | --- | --- | --- | --- | --- | --- | --- | --- | --- | --- | --- | --- | --- | --- | --- | --- | --- | --- | --- | --- | --- | --- | --- | --- | --- | --- | --- | --- | --- | --- | --- | --- | --- | --- | --- | --- | --- | --- | --- | --- | --- | --- | --- | --- | --- | --- | --- | --- | --- | --- | --- | --- | --- | --- | --- | --- | --- | --- | --- | --- | --- | --- | --- | --- | --- | --- | --- | --- | --- | --- | --- | --- | --- | --- | --- | --- | --- | --- | --- | --- | --- | --- | --- | --- | --- | --- | --- | --- | --- | --- | --- | --- | --- | --- | --- | --- | --- | --- | --- | --- | --- | --- | --- | --- | --- | --- | --- | --- | --- | --- | --- | --- | --- | --- | --- | --- | --- | --- | --- | --- | --- | --- | --- | --- | --- | --- | --- | --- | --- | --- | --- | --- | --- | --- | --- | --- | --- | --- | --- | --- | --- | --- | --- | --- | --- | --- | --- | --- | --- | --- | --- | --- | --- | --- | --- | --- | --- | --- | --- | --- | --- | --- | --- | --- | --- | --- | --- | --- | --- | --- | --- | --- | --- | --- | --- | --- | --- | --- | --- | --- | --- | --- | --- | --- | --- | --- | --- | --- | --- | --- | --- | --- | --- | --- | --- | --- | --- | --- | --- | --- | --- | --- | --- | --- | --- | --- | --- | --- | --- | --- | --- | --- | --- | --- | --- | --- | --- | --- | --- | --- | --- | --- | --- | --- | --- | --- | --- | --- | --- | --- | --- | --- | --- | --- | --- | --- | --- | --- | --- | --- | --- | --- | --- | --- | --- | --- | --- | --- | --- | --- | --- | --- | --- | --- | --- | --- | --- | --- | --- | --- | --- | --- | --- | --- | --- | --- | --- | --- | --- | --- | --- | --- | --- | --- | --- | --- | --- | --- | --- | --- | --- | --- | --- | --- | --- | --- | --- | --- | --- | --- | --- | --- | --- | --- | --- | --- | --- | --- | --- | --- | --- | --- | --- | --- | --- | --- | --- | --- | --- | --- | --- | --- | --- | --- | --- | --- | --- | --- | --- | --- | --- | --- | --- | --- | --- | --- | --- | --- | --- | --- | --- | --- | --- | --- | --- | --- | --- | --- | --- | --- | --- | --- | --- | --- | --- | --- | --- | --- | --- | --- | --- | --- | --- | --- | --- | --- | --- | --- | --- | --- | --- | --- | --- | --- | --- | --- | --- | --- | --- | --- | --- | --- | --- | --- |
|  |  |  |  |  |  |  |  |

**Supplementary Table S3.** Isotopic results for enamel and bone apatite analyzed from the Oupan kiln in Anhui Province, China.

| **Sample type** | **δ^13^C(‰)** | **δ^18^O(‰)** |
| --- | --- | --- |
| Femur | -8.5 | -8.6 |
| Rib | -8.9 | -9.7 |
| LM_1_ | -8.8 | -5.6 |
| LM_2_ | -7.0 | -6.6 |
| LM_3_ | -5.3 | -7.2 |

**Supplementary Table S4.** Sample information including skeletal elements, age estimation, and the number of isotopic serial samples analyzed in this study.

| Tooth/Bone Type | Number of samples | Approximate age of  development (in years) | Ref. |
| --- | --- | --- | --- |
| Femora | 1 | ~10 years before death | [^7-9^](#_ENREF_7) |
| Ribs | 1 | ~2-5 years before death |
| LM_1_ | 16 | 0.3-10 | [^10^](#_ENREF_10) |
| LM_2_ | 16 | 2.5-15.5 |
| LM_3_ | 17 | 8.5-22~23 |

**Supplementary Table S5.** The PCI and BPI of the femur and rib.

| Sample No. | PCI | BPI |
| --- | --- | --- |
| Femur | 4.1 | 0.4 |
| Rib | 3.8 | 0.5 |

**Supplementary Fig. S1.** X-ray diffraction diagram of femur and rib.


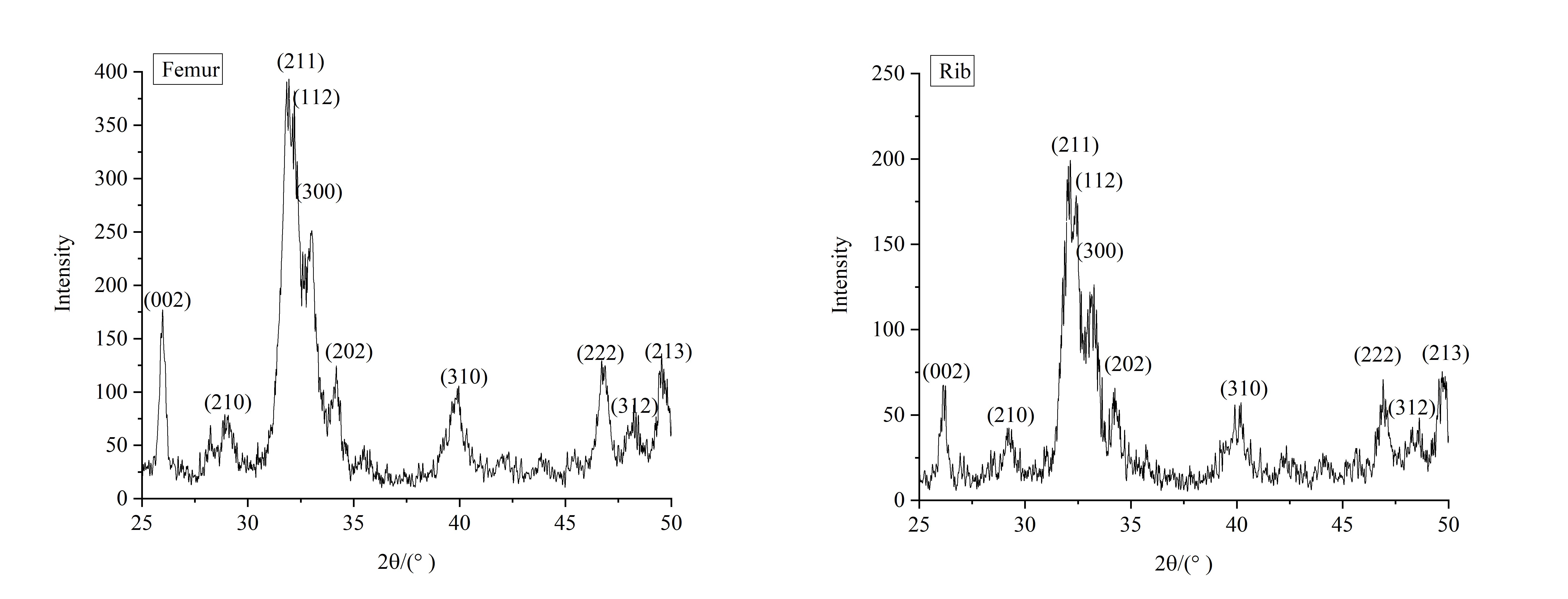


**Supplementary Fig. S2.** FTIR spectra of femur and rib.


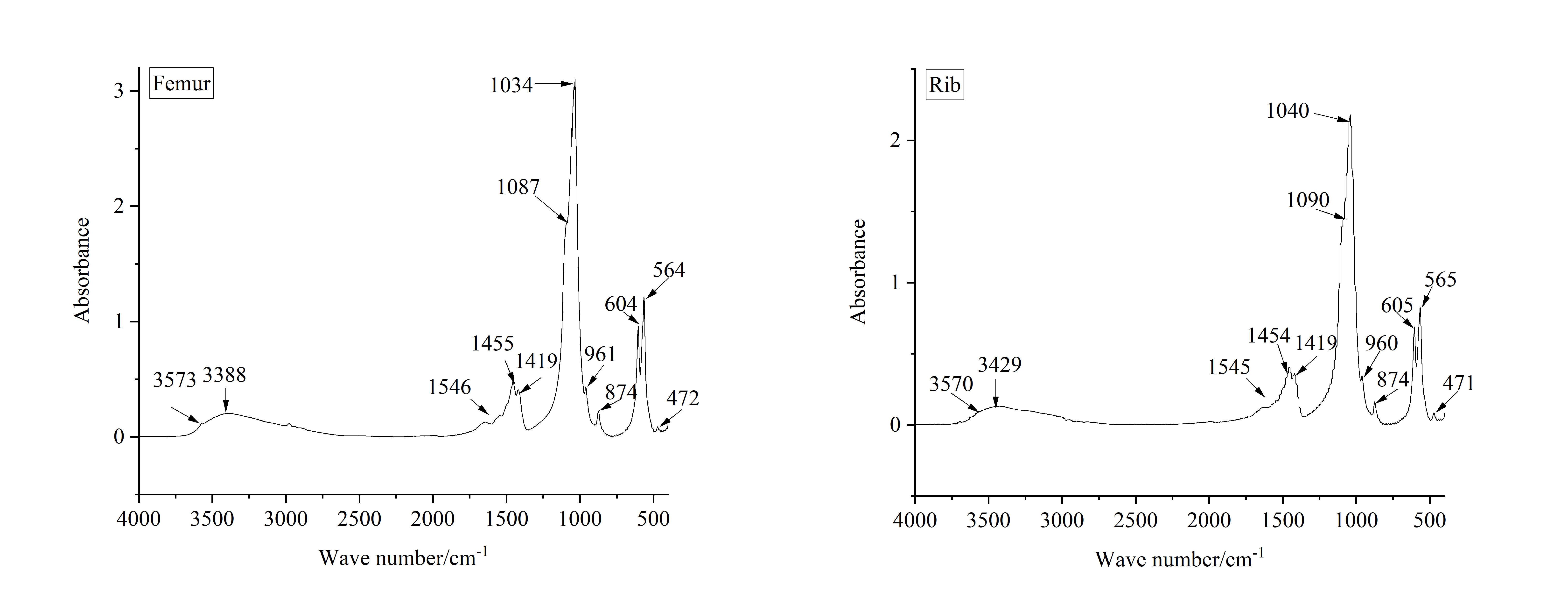


**References**

1 Bourne, G. H. *The biochemistry and physiology of bone*. (Elsevier, 2014).

2 Sillen, A. in *The chemistry of prehistoric human bone* (ed Wood B) 211-229 ( Cambridge University Press, 1989).

3 Sponheimer, M. & Lee-Thorp, J. A. Alteration of enamel carbonate environments during fossilization. *J. Archaeol. Sci.* **26**, 143-150 (1999).

4 Webb, E. C., White, C. D. & Longstaffe, F. J. Investigating inherent differences in isotopic composition between human bone and enamel bioapatite: implications for reconstructing residential histories. *J. Archaeol. Sci.* **50**, 97-107 (2014).

5 Shemesh, A. Crystallinity and diagenesis of sedimentary apatites. *Geochim. Cosmochim. Acta* **54**, 2433-2438 (1990).

6 Trueman, C. N., Privat, K. & Field, J. Why do crystallinity values fail to predict the extent of diagenetic alteration of bone mineral? *Paleogeogr. Paleoclimatol. Paleoecol.* **266**, 160-167 (2008).

7 Cox, G. & Sealy, J. Investigating Identity and Life Histories: Isotopic Analysis and Historical Documentation of Slave Skeletons Found on the Cape Town Foreshore, South Africa. *INT J HIST ARCHAEOL* **1**, 207-224, doi:10.1023/A:1027349115474 (1997).

8 Hedges, R. E. M., Clement, J. G., Thomas, C. D. L. & O'Connell, T. C. Collagen turnover in the adult femoral mid-shaft: Modeled from anthropogenic radiocarbon tracer measurements. *Amer. J. Phys. Anthrop.* **133**, 808-816, doi:10.1002/ajpa.20598 (2007).

9 Parfitt, A. M. Misconceptions (2): Turnover is always higher in cancellous than in cortical bone. *Bone* **30**, 807-809, doi:10.1016/S8756-3282(02)00735-4 (2002).

10 Beaumont, J. & Montgomery, J. Oral histories: A simple method of assigning chronological age to isotopic values from human dentine collagen. *Ann. Hum. Biol.* **42**, 405-412, doi:10.3109/03014460.2015.1045027 (2015).
